# Dead Again!

**Published:** 1892-09

**Authors:** 


DEAD AGAIN.—Poor little dickey-bird Fisher, of the *Southern Journal of Homeopathy*, is, as an editor (?) dead again. Old readers of the JOURNAL will remember how touchingly we related his early demise when as editor of the little *Pellet* he failed, and gave up his little homeopathic ghost with a "tit willow" gasp. He died then of an attack of DANIEL'S TEXAS MEDICAL JOURNAL, and he is dead of a relapse of the same malady now. He throws up the sponge convinced at last that he could never make a success as an *editor*. "Was it weakness of intellect, birdie, I cried, or a very tough worm on your little inside?" Both, doubtless—the red-back journal which never lets up on homeopathy, was worse than the tough worm, it was a Nessus shirt, and poor Fisher couldn't stand it. In the last issue of the *Southern Journal of Homeopathy* he chronicles his demise editorially, dying in orthodox theatrical style, taking four columns to expire in, after he is down;—like the villain in a blood and thunder play. The publishers are to be congratulated. The *Southern Journal of Homeopathy* is a pretty fair publication and has a wide field and ought to be made to pay; all that has been needed is an editor. Adieu, dickey-bird; peace to your little homeopathic ashes!

